# Clinical significance of hyaluronan levels and its pro-osteogenic effect on mesenchymal stromal cells in myelodysplastic syndromes

**DOI:** 10.1186/s12967-018-1614-4

**Published:** 2018-08-24

**Authors:** Cheng-Ming Fei, Juan Guo, You-Shan Zhao, Si-Da Zhao, Qing-Qing Zhen, Lei Shi, Xiao Li, Chun-Kang Chang

**Affiliations:** Department of Hematology, Shanghai Jiao Tong University Affiliated Sixth People’s Hospital, No. 600, Yi Shan Road, Shanghai, 200233 China

**Keywords:** Hyaluronan, Mesenchymal stromal cells, Osteogenic differentiation, Myelodysplastic syndromes

## Abstract

**Background:**

Hyaluronan (HA), a major component of the extracellular matrix, has been proven to play a crucial role in tumor progression. However, it remains unknown whether HA exerts any effects in myelodysplastic syndromes (MDS).

**Methods:**

A total of 82 patients with MDS and 28 healthy donors were investigated in this study. We firstly examined the bone marrow (BM) serum levels of HA in MDS by radioimmunoassay. Then we determined HA production and hyaluronan synthase (HAS) gene expression in BM mesenchymal stromal cells (MSC) and mononuclear cells derived from MDS patients. Finally, we investigated the effects of HA on osteogenic differentiation of MSC.

**Results:**

The BM serum levels of HA was increased in higher-risk MDS patients compared to normal controls. Meanwhile, patients with high BM serum HA levels had significantly shorter median survival than those with low HA levels. Moreover, the HA levels secreted by MSC was elevated in MDS, especially in higher-risk MDS. In addition, HAS-2 mRNA expression was also up-regulated in higher-risk MDS-MSC. Furthermore, we found that MSC derived from MDS patients with high BM serum HA levels had better osteogenic differentiation potential. Moreover, MSC cultured in HA-coated surface presented enhanced osteogenic differentiation ability.

**Conclusions:**

Our results show that elevated levels of BM serum HA are related to adverse clinical outcome in MDS. Better osteogenic differentiation of MSC induced by HA may be implicated in the pathogenesis of MDS.

## Background

Myelodysplastic syndromes (MDS) represent a diverse group of myeloid clonal disorders characterized by ineffective hematopoiesis, one or more cytopenias and potential progression to acute myeloid leukemia (AML). The precise mechanisms leading to the development of MDS are incompletely understood, however, the bone marrow (BM) niche may play an important role in the development, progression and response to treatment of MDS [1, 2]. The BM niche is mainly composed of mesenchymal stromal cells (MSC), endothelial cells, immune cells and other non-cell component such as various cytokines and extracellular matrix (ECM) [3].

Hyaluronan (HA), a major component of the ECM, is a member of the glycosaminoglycan polysaccharide family composed of repeating disaccharides of *N*-acetylglucosamine and glucuronic acid. HA is not only a structurally important molecule, but also has the potential to modify many cellular behaviors such as adhesion, proliferation, differentiation and migration [4, 5]. The effects of HA on cell behaviors are mediated by the HA receptors such as CD44, RHAMM, LYVE-1, layilin and HARE [6, 7]. In particular, HA production is increased in many malignant tissues and elevated HA levels have been shown to enhance tumor cell invasion, migration and proliferation [8, 9]. In addition, HA can be released by many cell types, both stromal cells and hematopoietic cells [10]. HA is expressed on human BM sinusoidal endothelium and endosteum, the regions where MSC are also abundant [11]. Recent research suggested that the HA could provide a protective niche for MSC, supporting the maintenance of their ‘stemness’ [12]. Several other reports supported a critical role of HA in the physiopathology of hematological malignancies, such as multiple myeloma (MM) [13, 14], AML [15]. However, it is still unclear whether HA exerts any effects in MDS.

In the present study we analyzed BM serum levels of HA from MDS patients and normal controls by radioimmunoassay, and concluded that higher-risk MDS patients had high BM serum levels of HA. The patients with high BM serum HA levels were correlated with poor prognosis. The HA production and hyaluronan synthase 2 (HAS-2) gene expression were elevated in higher-risk MDS-MSC. Moreover, we demonstrated that HA could facilitated osteogenic differentiation of MSC. MDS with high BM serum HA levels had better osteogenic differentiation potential of MSC. Our findings supported an important regulatory role for HA in the pathophysiology of MDS.

## Methods

### Patients

A total of 82 patients with MDS and 28 healthy donors from our own center between Jun 2011 and March 2014 were investigated in this study. All patients were untreated when they were recruited into this study. MDS was diagnosed in accordance with the minimum diagnostic criteria established by the conference on MDS (Vienna, 2006) [16].

### Measurement of the HA levels

The bone marrow serum and cell culture medium samples were centrifuged at 8000 g for 10 min. The supernatant was used to determine the total HA concentration. The HA levels were determined via radioimmunoassay [17].

### Isolation and culture of MSC

Following the isolation by density centrifugation, the BM mononuclear cells (MNC) were seeded at a concentration of 1 × 10^6^ cells/mL and cultured in Human Mesenchymal Stem Cell Growth Medium (Cyagen Biosciences Inc, Guangzhou, China) at 37 °C with 5% CO_2_ in a humidified atmosphere as previously described [18]. The supernatant containing non-adherent cells was removed and medium was changed every 3 days. MSC used in all experiments were derived from passages 2–4. To fulfill the criteria of the International Society for Cellular Therapy, MSC were evaluated by flow cytometry for the absence of CD34, CD45 antigens and the presence of CD73, CD90, CD105 and CD166 [19].

### Real-time quantitative polymerase chain reaction

RNA from MSC was purified using the RNeasy Mini Kit (QIAGEN, Germany) according to the manufacturer’s instructions. cDNA was prepared using the First Strand cDNA Synthesis Kit (Fermentas, Burlington, Canada) following the manufacturer’s protocol. PCR was performed on an ABI 7500 real-time PCR machine (Applied Biosystems). The primer sequences of runt related transcription factor 2 (RUNX-2), bone sialoprotein (BSP), alkaline phosphatase (ALP), Type1 collagen (COL-1), osteopontin (OPN), osteocalcin (OCN) and hyaluronan synthase 1/2/3 (HAS-1/2/3) are listed in Table 1. GAPDH served as reference control, and differences in mRNA expression levels were calculated as fold changes by the 2^−△△Ct^ method.

**Table 1:** The sequence of primers used for real time PCR

Prime	Forward (5′-3′)	Reverse (5′-3′)
GAPDH	CCCACTCCTCCACCTTTGA	CCACCCTGTTGCTGTAGCC
HAS-1	GGAATAACCTCTTGCAGCAGTTTC	TTGGGACCGCTGAAGCC
HAS-2	TCGCAACACGTAACGCAAT	ACTTCTCTTTTTCCACCCCATTT
HAS-3	AACAAGTACGACTCATGGATTTCCT	GCCCGCTCCACGTTGA
RUNX-2	AGTGGACGAGGCAAGAGTTTC	CCTTCTGGGTTCCCGAGGT
ALP	CCATTCCCACGTCTTCACATT	AAGGGCTTCTTGTCTGTGTCACT
COL-1	CACCAATCACCTGCGTACAGAA	CAGATCACGTCATCGCACAAC
BSP	GACAGTTCAGAAGAGGAGGAG	AGCCCAGTGTTGTAGCAGA
OCN	AGGGCAGCGAGGTAGTGAA	TCCTGAAAGCCGATGTGGT
OPN	TTTACAACAAATACCCAGATGC	ATGGCTTTCGTTGGACTTACT

### Osteogenic differentiation assay

MSC were seeded at 3 × 10^4^ cells/well in 6-well plate in Human Mesenchymal Stem Cell Osteogenic Differentiation Medium (Cyagen Biosciences Inc, Guangzhou, China), and the medium was replaced every 3 days. After 3 weeks differentiation, cells can be fixed and stained with Alizarin red, visualized using light microscopy. The bound staining was eluted with 10% (wt/vol) cetylpyridinium chloride (sigma), and alizarin red-S in samples was quantified by measuring absorbance at 572 nm.

### Cell culture with HA treatment

HA solution was prepared by dissolving HA powder (Mw = 1010 − 1800 KDa; LifeCore, MN, U.S.) in double distilled water and adjusted to working concentration before use. After the HA solution was applied to a 6-well plate surface, the coated substratum was kept to dried at 45 °C for 30 min. We used HA coated surfaces at a concentration of 30 μg/cm^2^.

### Statistical analysis

All statistical analyses were performed using the GraphPad Prism 5.01. The statistical differences between groups were determined by the two-tailed unpaired Student’s *t* test. Kaplan–Meier curves were used for analysis of overall survival (OS) and time to AML progression. The data were presented as mean ± SEM, p < 0.05 was considered statistically significant.

## Results

### Patient general features

In total, 82 patients were enrolled in this study. The follow-up cutoff date was defined as the end of June 2015. The median age was 58 years old (range 21–83 years), and the male-to-female ratio was 1.05:1. The patients were classified according to WHO 2016 [20] into MDS with single lineage dysplasia (MDS-SLD, n = 8), MDS with ring sideroblasts (MDS-RS, n = 4), MDS with multilineage dysplasia (MDS-MLD, n = 40), MDS with excess of blasts-1 (MDS-EB-1, n = 17), MDS with excess of blasts-2 (MDS-EB-2, n = 13). According to the IPSS risk category [21], 59 patients classified to higher-risk MDS (Intermediate-2/High, IPSS score ≥ 1.5) and 23 patients classified to lower-risk MDS (Low/Intermediate-1, IPSS score ≤ 1.0). Among the 82 patients, 54 patients had good karyotypes (including 51 normal karyotypes and 3 good abnormal karyotypes: sole 5q-, -y, and 20q-), 15 patients had intermediate risk abnormal karyotypes and 13 patients had poor risk abnormal karyotypes (Table 2).

**Table 2:** The clinical characteristics of all MDS patients

Characteristics	Values
Tested patients (n)	82
Median age (range)	58 (21–83)
Sex, n (%)	
Male	42 (51)
Female	40 (49)
WHO 2016 classification, n (%)	
MDS-SLD	8 10)
MDS-RS	4 (5)
MDS-MLD	40 (49)
MDS-EB-1	17 (20)
MDS-EB-2	13 (16)
IPSS risk groups, n (%)	
Low	5 (6)
Intermediate-1	54 (66)
Lower-risk	59 (72)
Intermediate-2	20 (24)
High	3 (4)
Higher-risk	23 (28)
Karyotypes by IPSS, n (%)	
Good	54 (66)
Intermediate	15 (18)
Poor	13 (16)

*WHO* World Health Organization, *MDS* myelodysplastic syndrome, *SLD* single lineage dysplasia, *RS* ring sideroblasts, *MLD* multilineage dysplasia, *EB* excess of blasts, *IPSS* International Prognostic Scoring System

### Levels of HA were elevated in BM serum of higher-risk MDS patients

**Fig. 1:**
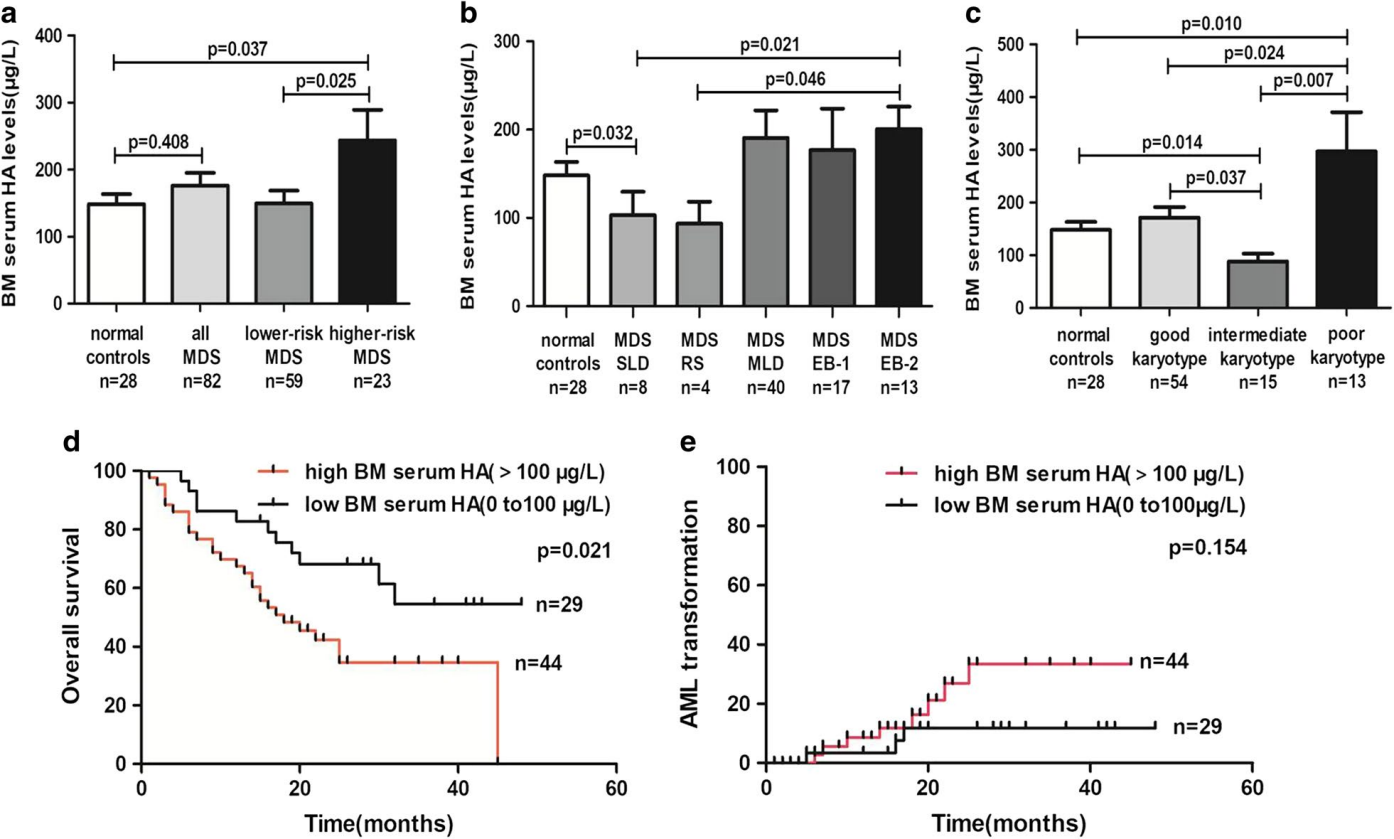
Clinical significance of BM serum HA levels in MDS. Comparion of BM serum HA levels between normal controls and **a** MDS sub-groups classified according to IPSS, **b** MDS sub-groups classified according to WHO, **c** MDS sub-groups classified according to karyotypes. **d** Overall survival and **e** time to AML progression in MDS patients with high and low levels of BM serum HA

Firstly we analyzed BM serum levels of HA from 82 MDS patients and 28 normal controls by radioimmunoassay. The mean HA levels of MDS patients was 176.1 μg/L (range from 18.1 to 800.0 μg/L), whereas the mean HA levels of normal controls was 148.2 μg/L (range from 22.3 to 304.2 μg/L). There was no obvious difference between MDS patients and normal controls (p = 0.408; Fig. 1a). According to the IPSS risk category, significantly elevated levels of HA were observed in higher-risk MDS patients compared to normal controls (243.5 ± 45.41 vs. 148.2 ± 15.07, p = 0.037), meanwhile,the levels of HA in higher-risk MDS patients were higher than those of lower-risk MDS patients (243.5 ± 45.41 vs. 149.8 ± 18.52, p = 0.025; Fig. 1a). Then we analyzed the HA levels in different WHO classification of MDS patients, the results showed that the HA levels in patients with MDS-SLD were significantly lower than normal controls (103.1 ± 26.41 vs. 148.2 ± 15.07, p = 0.032). The levels of HA in patients with MDS-EB-2 were higher than those of patients with MDS-SLD (200.6 ± 25.53 vs. 103.1 ± 26.41, p = 0.021) and MDS-RS (200.6 ± 25.53 vs. 93.7 ± 24.7, p = 0.046; Fig. 1b). Within karyotype categories, The HA levels in patients with poor karyotype were higher than normal controls (296.9 ± 74.34 vs. 148.2 ± 15.07, p = 0.01). However, the HA levels in patients with intermediate karyotype were lower than normal controls (88.26 ± 15.01 vs. 148.2 ± 15.07, p = 0.014) and patients with good karyotype (88.26 ± 15.01 vs. 171.4 ± 20.01, p = 0.037). Meanwhile, the patients with poor karyotype exhibited higher HA levels than those of patients with good and intermediate karyotype (296.9 ± 74.34 vs. 171.4 ± 20.01, p = 0.024; 296.9 ± 74.34 vs. 88.26 ± 15.01, p = 0.007, respectively; Fig. 1c). We also found that there was no obvious correlation between HA levels and percentage of BM blast cells (p = 0.45, r = 0.088).

### Patients with high BM serum HA levels had significantly shorter median survival

According to the BM serum levels of HA, all the patients were divided to two groups: high HA (> 100 μg/L) and low HA (0 to 100 μg/L). As shown in Fig. 1d, the median survival of patients with high HA levels was 18 months and the median survival of patients with low HA levels did not reach. The median survival between the two groups was statistically different (HR: 2.149, 95% CI 1.125 to 4.105, p = 0.021). However, no significant impact was seen on time to AML progression (HR: 2.372, 95% CI 0.724 to 7.772, p = 0.154; Fig. 1e).

### HA secreted by MSC was elevated in MDS, especially in higher-risk MDS

**Fig. 2:**
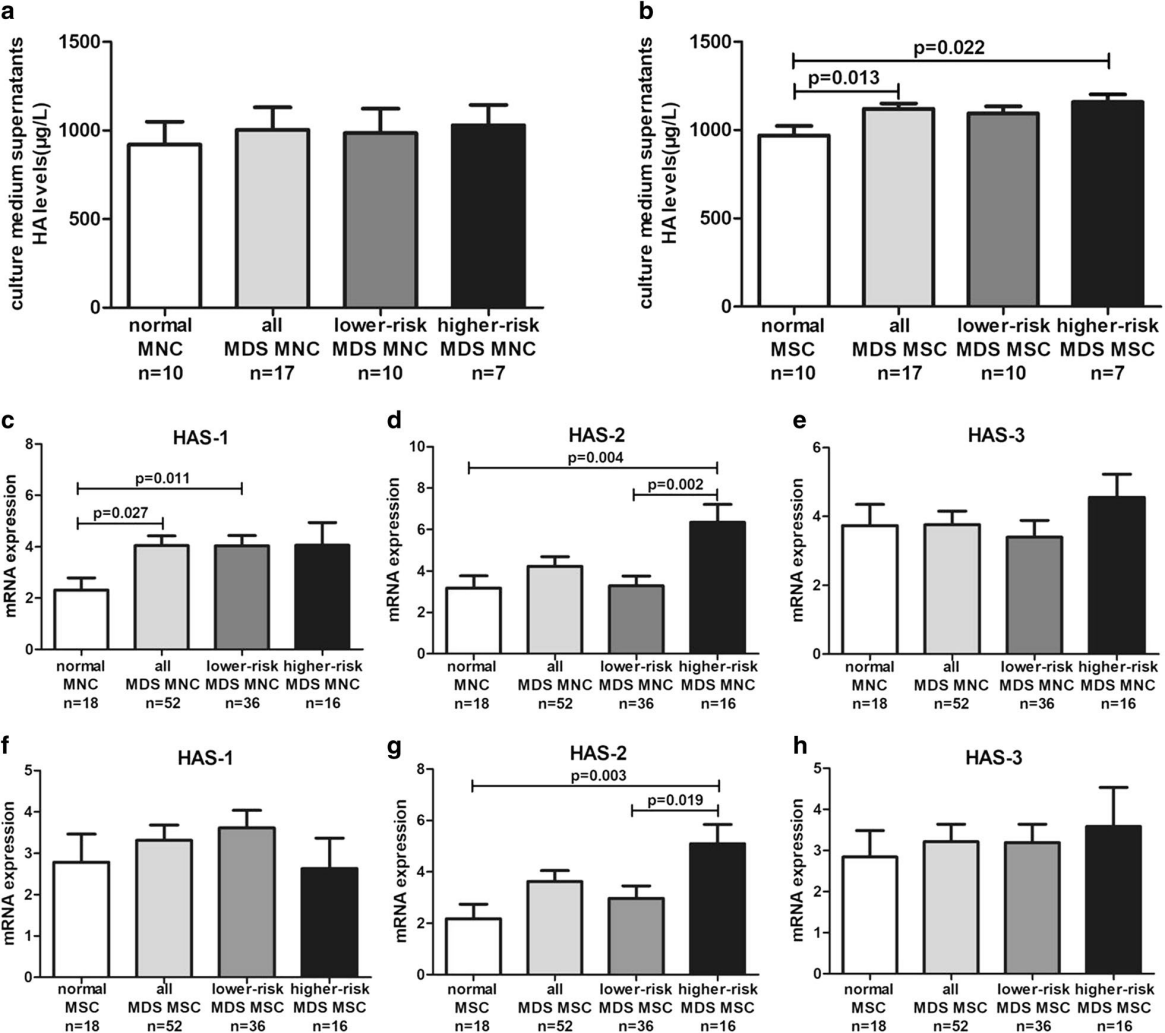
Comparion of HA production and HAS1/2/3 mRNA expression of MNC/MSC between normal controls and MDS. HA levels in culture medium supernatants of **a** MNC and **b** MSC. HAS1/2/3 mRNA expression in **c**–**e** MNC and **f**–**h** MSC

We next measured the HA levels in culture medium supernatants from MNC by radioimmunoassay. However, there was no difference between MDS patients and normal controls (Fig. 2a). We also examined HA levels in culture medium supernatants from MSC, we found that the HA levels were elevated in culture medium supernatants from MDS-MSC compared to normal controls (1121 ± 29.99 vs. 969.1 ± 54.12, p = 0.013), especially in higher-risk MDS-MSC (1159 ± 42.64 vs. 969.1 ± 54.12, p = 0.022; Fig. 2b). Furthermore, we determined the mRNA expression of HAS-1/2/3 in MDS-MNC. The results showed that the expression of HAS-1 in MDS-MNC was higher than normal controls (p = 0.027), and lower-risk MDS-MNC also exhibited significant higher HAS-1 expression than normal controls (p = 0.011; Fig. 2c). The expression of HAS-2 was up-regulated in higher-risk MDS-MNC compared to normal controls (p = 0.004) and lower-risk MDS-MNC (p = 0.002; Fig. 2d). No significant difference in HAS-3 expression was observed between the MNC of normal controls and MDS patients (Fig. 2e). We also investigated the mRNA expression of HAS-1/2/3 in MDS-MSC. HAS-2 mRNA expression was 2.3-fold greater in higher-risk MDS-MSC compared with normal MSC (p = 0.003), meanwhile, HAS-2 mRNA expression was significantly increased in higher-risk MDS-MSC compared to lower-risk MDS-MSC (p = 0.019; Fig. 2g). However, there was no difference between any groups in HAS-1 or HAS-3 (Fig. 2f, h).

### MDS with high BM serum HA levels had better osteogenic differentiation function in MSC

**Fig. 3:**
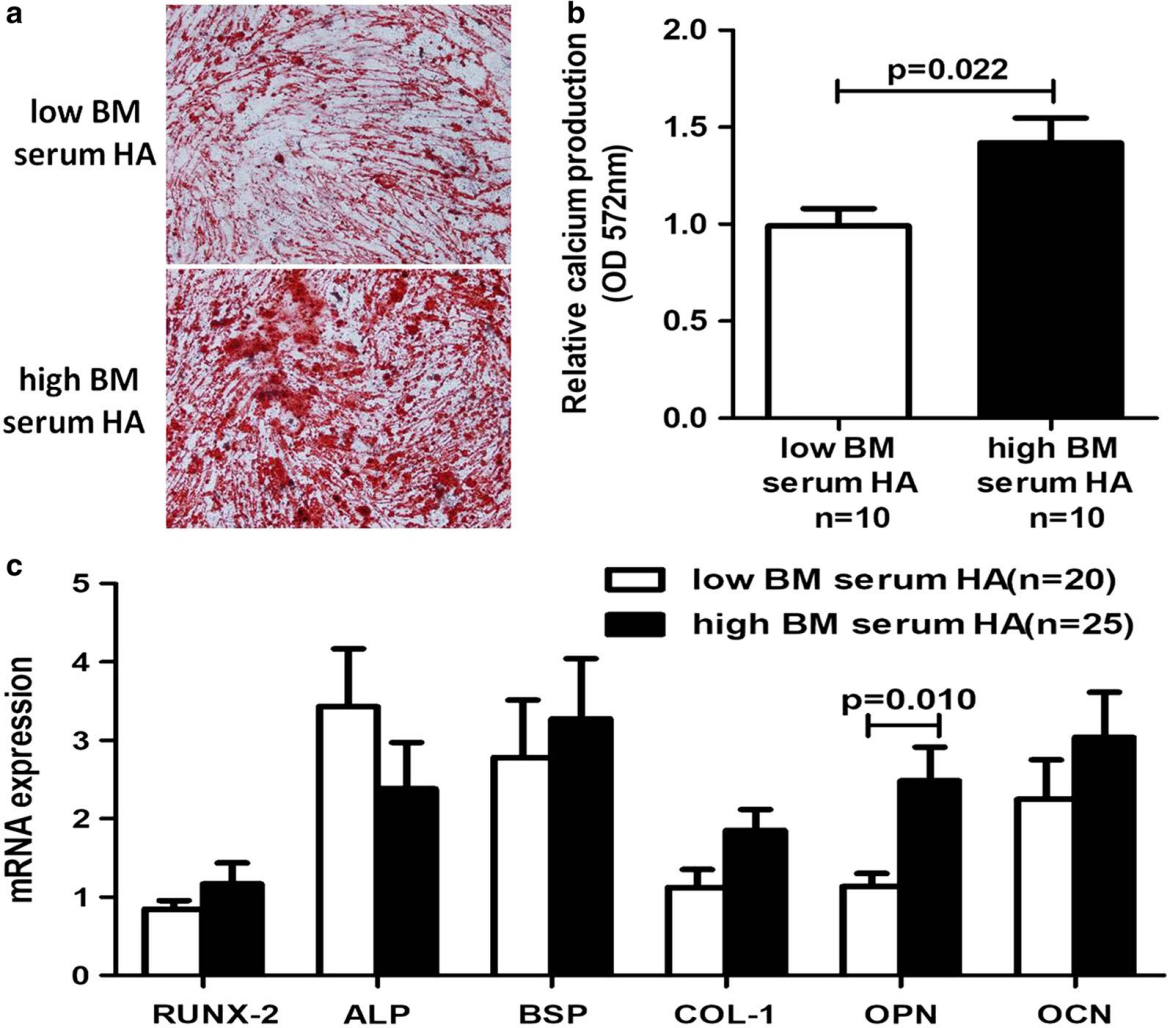
Osteogenic differentiation potential of MSC from MDS patients with high and low BM serum HA levels. **a** After 21 days in osteogenic medium, representative micrographs of Alizarin Red S staining of MDS patients with high and low BM serum HA levels are shown. Red staining highlights deposition of mineralized extracellular matrix by differentiated MSC. Magnification: 10×. **b** Differences with regard to osteogenic differentiation were quantified by the OD value. **c** Comparion of mRNA expression of osteogenic differentiation related genes (RUNX-2, BSP, ALP, COL-1, OPN, OCN) between MDS patients with high and low BM serum HA levels

In a next step we selected ten patients with high BM serum levels of HA and ten patients with low BM serum levels of HA,then investigate the osteogenic differentiation potential of MSC between two groups. After 21 days of osteogenic induction differentiation, relative calcium production by MSC from MDS patients with low BM serum levels of HA was reduced compared to MDS patients with high BM serum levels of HA (p = 0.022; Fig. 3a, b). In addition, mRNA expression of genes associated with osteogenic differentiation in MSC was investigated, the results showed that OPN mRNA expression was significantly increased in MSC from patients with high BM serum levels of HA (p = 0.010). However, the mRNA expression of the other genes (RUNX-2, BSP, ALP, COL-1, OCN) investigated were no obvious difference between two groups (Fig. 3c).

### HA induced MSC osteogenic differentiation

**Fig. 4:**
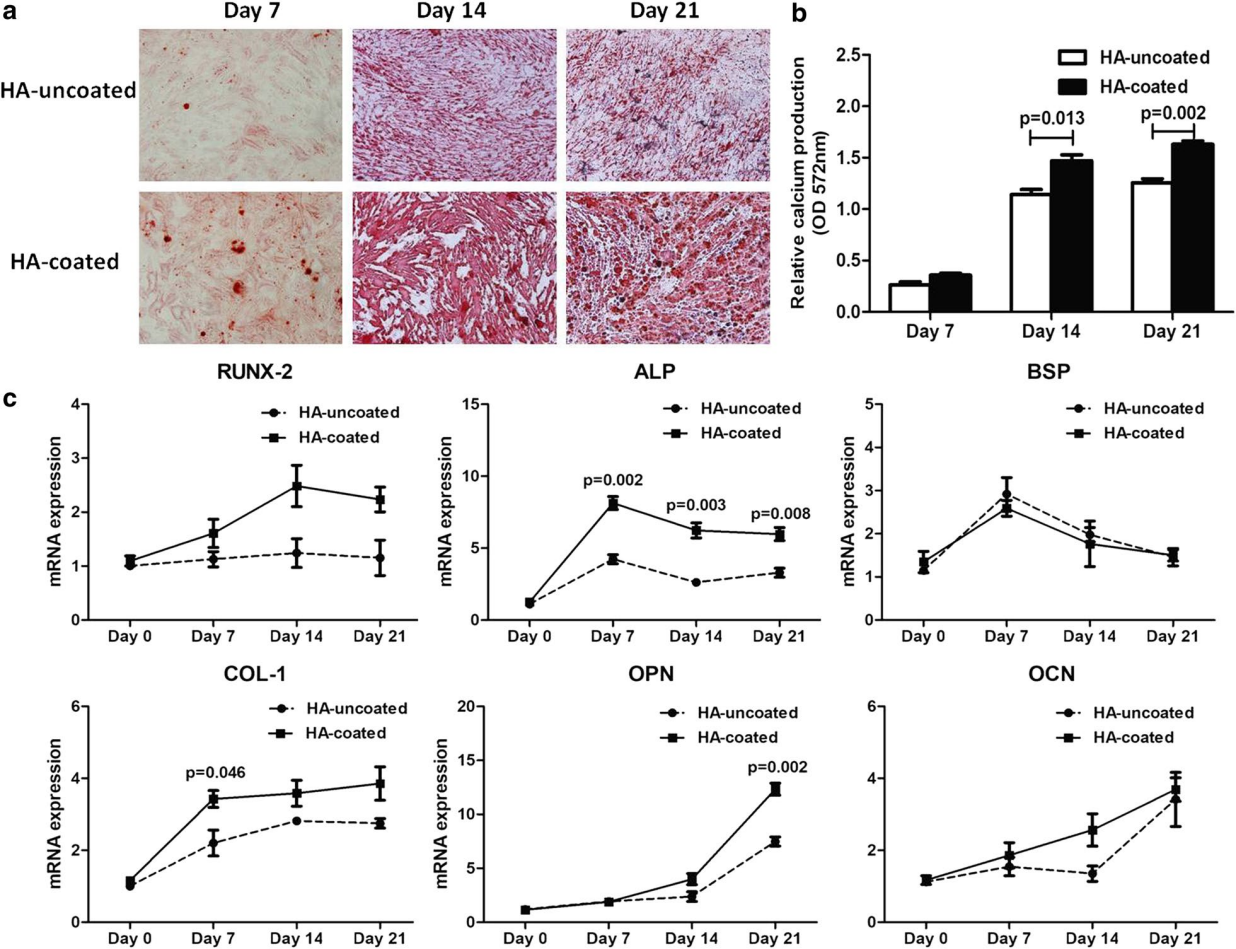
Effect of HA on osteogenic differentiation of MSC. **a** Osteogenic induction assay of MSC on surface with and without HA-coating was evaluated at day 7, day 14 and day 21 respectively. Representative micrographs of Alizarin Red S staining of HA-uncoated MSC and HA-coated MSC are shown. Magnification: 10×. **b** Differences with regard to osteogenic differentiation were quantified by the OD value. **c** Comparion of mRNA expression of osteogenic differentiation related genes (RUNX-2, BSP, ALP, COL-1, OPN, OCN) between HA-uncoated group and HA-coated group

Finally, to further study the involvement of HA in the regulation of osteogenic differentiation of MSC, we performed an osteogenic induction culture of MSC with low BM serum HA levels on surface with and without HA-coating, and evaluated the mineralization at day 7, 14, and 21, respectively. As shown in Fig. 4a, mineralization was evident from day 14, which was confirmed by alizarin red S staining. Compared with normal controls, the relative calcium production increased in MSC with HA-coated surface at day 14 and day 21 (p = 0.013, p = 0.002, respectively; Fig. 4b). Next we investigated the expression of genes associated with osteogenic differentiation in MSC following osteogenic induction at day 0, 7, 14, and 21. MSC with HA-coated demonstrated higher ALP gene expression compared with normal controls at each of the time points (day 7, p = 0.002; day 14, p = 0.003; day 21, p = 0.008, respectively). The results also showed that the expression levels of COL-1 was increased in MSC with HA-coated at day 7 compared to normal controls (p = 0.046). Moreover, MSC with HA-coated showed the higher OPN expression than normal controls at day 21 (p = 0.002; Fig. 4c).

## Discussion

There is increasing evidence that HA production is elevated in tumors and may play an important role in tumor progression [9, 22, 23]. However, the clinical implication of HA levels in MDS remains unclear. HA has been always detected in various body fluids, such as serum, lymph, urine, and pleural fluid [24]. To better understand the effect of HA in the BM niche of MDS, we examined the HA levels in bone marrow serum of MDS patients in this study. The main finding was that higher-risk MDS patients showed significantly elevated BM serum HA levels, as compared to normal controls. Earlier study evaluated the HA distribution in AML/MDS by histochemical stain,and showed that AML patients exhibited stronger HA staining, four of 8 MDS patients showed higher HA staining compared to normal controls [15]. An interesting observation in the present study is that an inverse correlation between OS and BM serum HA levels. However, BM serum HA levels were not associated with time to AML progression. In addition, the cell surface receptor, CD44 has been shown to be important in malignant cell adhesion, survival, migration, and invasion. Serum CD44 was reported slightly increased in MDS patients [25]. Another study also demonstrated that elevated serum CD44  levels were associated with shorter survival in MDS patients [26]. These results indicate that HA-CD44 signals are activated in MDS, suggesting their critical role in the pathogenesis of MDS.

Elevated BM serum HA levels which may be caused by increased HA production by the malignant cells themselves or surrounding stromal cells. Moreover, HA biosynthesis is regulated by three transmembrane glycosyltransferase isoenzymes: HAS-1, HAS-2 and HAS-3 [27]. Therefore, we characterized HA production and HAS-1/2/3 gene expression in MNC and MSC. We examined HA levels in culture medium supernatants from MSC,we have shown that the levels of HA secreted by MSC was increased in MDS, especially in higher-risk MDS, up-regulated mRNA expression of HAS-2 in higher-risk MDS-MSC might explain the overproduction of HA. In addition, we also found the HA could secret by MNC, although the MDS-MNC exhibited significant higher mRNA expression of HAS-1/2,this did not affect the HA production in MNC from MDS patients. All these data indicate that MSC play a prominent role in the augmentation of HA levels in the BM serum of higher-risk MDS.

It is well known that lower-risk MDS and higher-risk MDS possess different biological characteristics, lower-risk MDS present a trend to BM failure, while higher-risk MDS are more likely to convert into leukemia. In this study we found the obvious differences in the BM serum levels of HA between lower-risk and higher-risk MDS. Our previous study also showed the differences in osteogenic state between lower-risk and higher-risk MDS-MSC, the osteogenic differentiation potential of lower-risk MDS-MSC were impaired,but those of higher-risk MDS-MSC were relatively normal [18]. Thus we speculate that the distinct HA levels in lower-risk and higher-risk MDS may participate in these processes. We then separately analyzed osteogenic differentiation potential of MSC from patients with high and low BM serum HA levels. Interestingly, MDS with high HA levels exhibited better osteogenic differentiation potential of MSC. These results indicate that HA may assotiated with pro-osteogenic differentiation potential of MSC in MDS. Osteoblasts, as major stromal cells derived from MSC in the endosteal niche, support hematopoietic progenitors and cause secretion of several cytokines. High osteoblast activity in MDS BM niche may facilitate the growth of malignant cells [28]. Therefore, it may be that perturbations of osteoblast activity drive MDS pathogenesis.

Futhermore, in order to confirm the effects of HA on MSC osteogenic differentiation, we performed osteogenic induction culture of MSC on surface with and without HA-coating. The results showed that HA could enhance the osteogenic differentiation function of MSC. The mechanism by which HA exerts its pro-osteogenic effect on MSC is still poorly defined. Several studies demonstrated that HA presented pro-osteogenic differentiation via stimulated expression of specific target genes such as ALP, osterix, RUNX-2, COL-1 or enhanced cell adhesion functions [29–31]. In addition, HA may act as a reservoir for the growth factors which reported to be important in osteogenesis, such as platelet-derived growth factor (PDGF), different bone morphogenetic proteins (BMPs) and transforming growth factor-β (TGF-β) [32, 33]. These are possible mechanisms that may account for its osteogenesis regulatory properties. However, Chang et al. [34] also demonstrated that HA could inhibit osteogenic differentiation through TLR4 by interfering with M-CSF. The discrepancies between the reports may be attributed to the different molecular weight and concentration of HA investigated.

## Conclusions

Our results showed that higher-risk MDS patients had increased BM serum levels of HA, and high BM serum levels of HA may be of prognostic value in MDS. Overproduction of HA from MSC had a pro-osteogenic effect and may participate in MDS pathogenesis.

## Abbreviations

HA: hyaluronan
ECM: extracellular matrix
MDS: myelodysplastic syndromes
AML: acute myeloid leukemia
BM: bone marrow
OS: overall survival
MSC: mesenchymal stromal cells
MNC: mononuclear cells
SLD: single lineage dysplasia
RS: ring sideroblasts
MLD: multilineage dysplasia
EB: excess of blasts
IPSS: International Prognostic Scoring System
HAS: hyaluronan synthase
RUNX-2: runt related transcription factor2
BSP: bone sialoprotein
ALP: alkaline phosphatase
COL-1: Type1 collagen
OPN: osteopontin
OCN: osteocalcin

## References

[1] Li AJ, Calvi LM (2017). The microenvironment in myelodysplastic syndromes: niche-mediated disease initiation and progression. Exp Hematol.

[2] Raaijmakers MH (2012). Myelodysplastic syndromes: revisiting the role of the bone marrow microenvironment in disease pathogenesis. Int J Hematol.

[3] Bonnans C, Chou J, Werb Z (2014). Remodelling the extracellular matrix in development and disease. Nat Rev Mol Cell Biol.

[4] Vigetti D, Karousou E, Viola M (2014). Hyaluronan: biosynthesis and signaling. Biochim Biophys Acta.

[5] Solis MA, Chen YH, Wong TY (2012). Hyaluronan regulates cell behavior: a potential niche matrix for stem cells. Biochem Res Int.

[6] Misra S, Hascall VC, Markwald RR (2015). Interactions between hyaluronan and its receptors (CD44, RHAMM) regulate the activities of inflammation and cancer. Front Immunol.

[7] Zhou B, Weigel JA, Fauss L (2000). Identification of the hyaluronan receptor for endocytosis (HARE). J Biol Chem.

[8] Shigeeda W, Shibazaki M, Yasuhira S (2017). Hyaluronic acid enhances cell migration and invasion via the YAP1/TAZ-RHAMM axis in malignant pleural mesothelioma. Oncotarget.

[9] Schwertfeger KL, Cowman MK, Telmer PG (2015). Hyaluronan, Inflammation, and breast cancer progression. Front Immunol.

[10] Kota DJ, Prabhakara KS, Cox CS (2014). MSCs and hyaluronan: sticking together for new therapeutic potential?. Int J Biochem Cell Biol.

[11] Avigdor A, Goichberg P, Shivtiel S (2004). CD44 and hyaluronic acid cooperate with SDF-1 in the trafficking of human CD34+ stem/progenitor cells to bone marrow. Blood.

[12] Qu C, Rilla K, Tammi R (2014). Extensive CD44-dependent hyaluronan coats on human bone marrow-derived mesenchymal stem cells produced by hyaluronan synthases HAS1, HAS2 and HAS3. Int J Biochem Cell Biol.

[13] Dahl IM, Turesson I, Holmberg E (1999). Serum hyaluronan in patients with multiple myeloma: correlation with survival and Ig concentration. Blood.

[14] Vincent T, Molina L, Espert L (2003). Hyaluronan, a major non-protein glycosaminoglycan component of the extracellular matrix in human bone marrow, mediates dexamethasone resistance in multiple myeloma. Br J Haematol.

[15] Sundstrom G, Dahl IM, Hultdin M (2005). Bone marrow hyaluronan distribution in patients with acute myeloid leukemia. Med Oncol.

[16] Valent P, Horny HP, Bennett JM (2007). Definitions and standards in the diagnosis and treatment of the myelodysplastic syndromes: consensus statements and report from a working conference. Leuk Res.

[17] Wu M, Cao M, He Y (2015). A novel role of low molecular weight hyaluronan in breast cancer metastasis. FASEB J.

[18] Fei C, Zhao Y, Gu S (2014). Impaired osteogenic differentiation of mesenchymal stem cells derived from bone marrow of patients with lower-risk myelodysplastic syndromes. Tumour Biol.

[19] Dominici M, Le Blanc K, Mueller I (2006). Minimal criteria for defining multipotent mesenchymal stromal cells. The International Society for Cellular Therapy position statement. Cytotherapy.

[20] Arber DA, Orazi A, Hasserjian R (2016). The 2016 revision to the World Health Organization classification of myeloid neoplasms and acute leukemia. Blood.

[21] Greenberg P, Cox C, LeBeau MM (1997). International scoring system for evaluating prognosis in myelodysplastic syndromes. Blood.

[22] Chanmee T, Ontong P, Itano N (2016). Hyaluronan: a modulator of the tumor microenvironment. Cancer Lett.

[23] Itano N, Zhuo L, Kimata K (2008). Impact of the hyaluronan-rich tumor microenvironment on cancer initiation and progression. Cancer Sci.

[24] Cowman MK, Lee HG, Schwertfeger KL (2015). The content and size of hyaluronan in biological fluids and tissues. Front Immunol.

[25] Nasu H, Hibi N, Ohyashiki JH (1998). Serum soluble CD44 levels for monitoring disease states in acute leukemia and myelodysplastic syndromes. Int J Oncol.

[26] Loeffler-Ragg J, Steurer M, Ulmer H (2006). Elevated levels of serum CD44 and E-cadherin predict an unfavourable outcome in myelodysplastic syndromes. Leukemia.

[27] Tammi RH, Passi AG, Rilla K (2011). Transcriptional and post-translational regulation of hyaluronan synthesis. FEBS J.

[28] Cogle CR, Saki N, Khodadi E (2015). Bone marrow microenvironment in the myelodysplastic syndromes. Leuk Res.

[29] Kliemt S, Lange C, Otto W (2013). Sulfated hyaluronan containing collagen matrices enhance cell-matrix-interaction, endocytosis, and osteogenic differentiation of human mesenchymal stromal cells. J Proteome Res.

[30] Mathews S, Mathew SA, Gupta PK (2014). Glycosaminoglycans enhance osteoblast differentiation of bone marrow derived human mesenchymal stem cells. J Tissue Eng Regen Med.

[31] Jha AK, Xu X, Duncan RL (2011). Controlling the adhesion and differentiation of mesenchymal stem cells using hyaluronic acid-based, doubly crosslinked networks. Biomaterials.

[32] Chen G, Deng C, Li YP (2012). TGF-beta and BMP signaling in osteoblast differentiation and bone formation. Int J Biol Sci.

[33] Astachov L, Vago R, Aviv M (2011). Hyaluronan and mesenchymal stem cells: from germ layer to cartilage and bone. Front Biosci.

[34] Chang EJ, Kim HJ, Ha J (2007). Hyaluronan inhibits osteoclast differentiation via Toll-like receptor 4. J Cell Sci.

